# Age at first sexual intercourse and frailty: Mendelian randomization and population-based evidence from NHANES and UK Biobank

**DOI:** 10.1093/sexmed/qfag075

**Published:** 2026-09-02

**Authors:** Kai-Xian Wang, Bao-Peng Liu, Jia-Ning Wang, Kai-Zheng Wang, Yi-Zhan He, Meng-Yao Yu, Zi-Ming Shao, Long Sun, Mei-Xia Xu

**Affiliations:** Department of Social Medicine and Health Management, School of Public Health, Cheeloo College of Medicine, Shandong University, Jinan 250012, China; National Health Commission of China (NHC) Key Lab of Health Economics and Policy Research (Shandong University), Jinan 250012, China; Center for Health Management and Policy Research, Shandong University (Shandong Provincial Key New Think Tank), Jinan 250012, China; School of Psychiatry and Psychology, Cheeloo College of Medicine, Shandong University, Jinan 250012, China; Department of Epidemiology, School of Public Health, Cheeloo College of Medicine, Shandong University, Jinan 250012, China; Department of Epidemiology, School of Public Health, Cheeloo College of Medicine, Shandong University, Jinan 250012, China; School of Acupuncture and Tuina, Shandong University of Traditional Chinese Medicine, Jinan 250355, China; Department of Social Medicine and Health Management, School of Public Health, Cheeloo College of Medicine, Shandong University, Jinan 250012, China; National Health Commission of China (NHC) Key Lab of Health Economics and Policy Research (Shandong University), Jinan 250012, China; Center for Health Management and Policy Research, Shandong University (Shandong Provincial Key New Think Tank), Jinan 250012, China; School of Psychiatry and Psychology, Cheeloo College of Medicine, Shandong University, Jinan 250012, China; Department of Social Medicine and Health Management, School of Public Health, Cheeloo College of Medicine, Shandong University, Jinan 250012, China; National Health Commission of China (NHC) Key Lab of Health Economics and Policy Research (Shandong University), Jinan 250012, China; Center for Health Management and Policy Research, Shandong University (Shandong Provincial Key New Think Tank), Jinan 250012, China; School of Psychiatry and Psychology, Cheeloo College of Medicine, Shandong University, Jinan 250012, China; Department of Social Medicine and Health Management, School of Public Health, Cheeloo College of Medicine, Shandong University, Jinan 250012, China; National Health Commission of China (NHC) Key Lab of Health Economics and Policy Research (Shandong University), Jinan 250012, China; Center for Health Management and Policy Research, Shandong University (Shandong Provincial Key New Think Tank), Jinan 250012, China; School of Psychiatry and Psychology, Cheeloo College of Medicine, Shandong University, Jinan 250012, China; Department of Social Medicine and Health Management, School of Public Health, Cheeloo College of Medicine, Shandong University, Jinan 250012, China; National Health Commission of China (NHC) Key Lab of Health Economics and Policy Research (Shandong University), Jinan 250012, China; Center for Health Management and Policy Research, Shandong University (Shandong Provincial Key New Think Tank), Jinan 250012, China; School of Psychiatry and Psychology, Cheeloo College of Medicine, Shandong University, Jinan 250012, China; School of Marxism, Shandong Women’s University, Jinan 250300, China

**Keywords:** age at first sexual intercourse, frailty, National Health and Nutrition Examination Survey, UK Biobank, Mendelian randomization

## Abstract

**Introduction:**

Age at first sexual intercourse (AFS) is an indicator of sexual initiation and may reflect broader life-course trajectories, but its relationship with frailty remains unclear. To examine genetic evidence for a potential association between AFS and frailty and to characterize nonlinear, threshold, and sex-specific patterns in population-based observational analyses.

**Methods:**

We performed Mendelian randomization (MR) analyses using summary statistics for AFS and frailty. We further analyzed data from the National Health and Nutrition Examination Survey (NHANES; *n* = 8282) and UK Biobank (UKB; *n* = 109 449) among participants aged 50 years or older with AFS ≥15 years. Participants reporting AFS <15 years were analyzed separately. Frailty status was defined using a frailty index cutoff of >0.21, and the nonlinear and threshold associations between AFS and frailty.

**Results:**

MR analyses showed that genetically predicted earlier AFS was associated with higher frailty risk (β = -0.30; 95% CI, -0.36 to -0.25), with similar estimates in males (β = -0.23; 95% CI, -0.30 to -0.16) and females (β = -0.22; 95% CI, -0.30 to -0.13). In observational analyses, restricted cubic spline models showed significant L-shaped associations between AFS and frailty in both NHANES and UKB. AFS <15 years was not significantly associated with frailty in the overall analyses. Compared with the third quintile of AFS, participants in the first and second quintiles had 61% (OR = 1.61, 95% CI: 1.40-1.85) and 19% (OR = 1.19, 95% CI, 1.05-1.41) higher odds of frailty, respectively, whereas fifth quintiles had 23% (OR = 0.77, 95% CI, 0.64-0.91) lower odds of frailty in NHANES; participants in the first quintile had 34% (OR = 1.34, 95% CI, 1.18-1.52) higher odds of frailty in UKB. Threshold analyses identified integer cut points of 21 years in NHANES and 23 years in UKB. Below these thresholds, later AFS was associated with lower odds of frailty; above them, associations were not statistically significant.

**Conclusion:**

MR analyses provided genetic evidence consistent with a potential association between earlier AFS and higher frailty risk, while population-based analyses showed an L-shaped association among middle-aged and older adults. These findings should be interpreted cautiously and not as definitive evidence of causality. Earlier AFS may be relevant to frailty risk later in life, supporting safe, informed, and rights-based sexual development rather than defining an optimal age for sexual debut.

## Background

Age at first sexual intercourse (AFS) is a common indicator of sexual initiation and may reflect broader life-course context. This is partly because the timing of first sexual intercourse intersects with individuals’ long-term development,^1^ sexual and reproductive health,^2^ and risk of adverse outcomes.^3^ Earlier AFS, particularly in the absence of adequate protection and support, has been associated with a range of adverse outcomes, including adolescent pregnancy,^2^ sexually transmitted infections,^4^ and mental health problems.^3^^,^^5^ At the same time, AFS should not be interpreted simplistically as a marker of either poor or good health, because it captures the timing of sexual debut rather than the broader relational, social, and consent-related context of first sex. In this sense, AFS may be more appropriately understood as a marker of life-course context and trajectory.

With increasing longevity, frailty has become a major public health concern in aging societies.^6^ Frailty is commonly characterized by diminished physiological reserve and heightened vulnerability to stressors, and it is particularly relevant in middle-aged and older adults. Recent reviews suggest that frailty affects roughly 12%-24% of older adults globally.^7^ Frailty is clinically important because it is associated with a wide range of adverse outcomes, including mortality,^8^ mental health burden,^9^ and economic burden.^10^ Previous studies on frailty have mainly focused on more established determinants, including sociodemographic disadvantage, multimorbidity, malnutrition, low physical activity, depression, sleep problems, cognitive impairment, and social vulnerability.^11–13^ By contrast, AFS has received much less direct attention as a potential life-course determinant of frailty, despite emerging evidence suggesting that frailty may be shaped by exposures operating well before older age.^11^

From a life-course perspective, AFS may relate to frailty in midlife and later life. Prior Mendelian randomization (MR) research has suggested that genetically predicted female reproductive traits, including AFS, may be associated with frailty.^14^ One possible explanation is that later AFS in women may reflect a more stable physiological pattern of estrogen exposure, thereby reducing muscle loss and decline in physiological reserve caused by hormonal dysregulation and lowering frailty risk.^14^ However, it remains unclear whether this association differs by sex or shows nonlinear patterns with identifiable inflection points in population-based settings.

The shape of the AFS-frailty relationship may be important because first sex at different ages may reflect different developmental stages, psychosocial maturity, and access to protective resources. The association may therefore be stronger at younger ages and attenuate beyond a certain point. Any identified threshold should be interpreted as a statistical point where the association changes, not as an optimal or recommended age for sexual debut.

To address these gaps, the present study first used MR to examine genetic evidence for a potential causal association between AFS and frailty and to assess whether this association differs by sex. We then analyzed data from two large population-based studies, the National Health and Nutrition Examination Survey (NHANES) and the UK Biobank (UKB), to further examine the association between AFS and frailty among middle-aged and older adults, with particular attention to nonlinear patterns, age ranges in which the strength of association may shift, and sex differences. Accordingly, the present study was designed to address the following question: Is earlier AFS associated with higher frailty risk, and is this association supported by MR evidence and characterized by nonlinear or sex-specific patterns in large population-based studies?

## Methods

### Study design

This study integrated MR and population-based observational analyses. MR was used to examine genetic evidence for a potential AFS-frailty association and sex-specific patterns. NHANES and UKB were then used to assess nonlinear associations, threshold ranges, and sex differences among middle-aged and older adults.

### Subject population

NHANES is an ongoing study conducted by the Centers for Disease Control and Prevention in the United States, providing estimates of the lifestyle, health, and nutritional status of the US civilian population. We conducted a secondary analysis of nine independent 2-year NHANES survey cycles from1999-2000 to 2015-2016. The UKB recruited over 500 000 participants aged 37-73 years from 22 centers across England, Scotland, and Wales between 2006 and 2010. Details of the study design and data collection have been described previously.^15^ Because frailty is primarily an age-related syndrome and is more clinically meaningful in middle-aged and older populations, participants younger than 50 years were excluded from the observational analyses. Participants with missing AFS or frailty data were also excluded. Ultimately, 8282 participants from NHANES and 109 449 participants from UKB were included in the primary observational analyses among participants with AFS ≥15 years (Supplementary Figures 1 and 2). Participants reporting AFS <15 years were analyzed separately as a distinct very early AFS group.

### Measures

#### Data acquisition of MR analysis

Summary statistics for AFS were obtained from the largest Genome-wide association studies (GWAS) meta-analysis.^16^ Summary statistics for frailty were obtained from a GWAS meta-analysis of UKB and the Swedish Twin-Gene cohort, including 175 226 participants of European ancestry.^17^

#### Assessment of AFS

AFS was assessed using self-reported age at first sex (including vaginal, anal, or oral sex). Because very early reported AFS may reflect childhood sexual abuse, coercion, statutory concerns, or other adverse contexts, AFS <15 years was not treated as ordinary sexual debut in the primary analyses. Instead, the primary quintile-based, restricted cubic spline (RCS), and threshold analyses were conducted among participants with AFS ≥15 years in both cohorts. Participants reporting AFS <15 years were analyzed separately as a distinct very early AFS group.

#### Assessment of frailty

Frailty was operationalized using the frailty index (FI). In NHANES, FI was defined according to the criteria proposed by Hakeem et al,^18^ encompassing 49 items across multiple domains, including cognitive function, dependence, depressive symptoms, comorbidities, hospital utilization and access to care, laboratory indicators, and physical performance. FI was calculated as the ratio of deficits present to the total number of possible deficits, yielding a continuous score ranging from 0 to 1, with higher scores indicating higher levels of frailty.^19^ Participants were categorized as non-frail or frail using the established cutoff of FI >0.21.^19^ In UKB, 49 self-reported deficit items, including signs, symptoms, and diseases across physiological and mental domains, were selected to construct the FI.^20^ Participants were excluded if they had missing data for 10 or more items used to calculate the FI. FI was calculated as the sum of deficits divided by the number of non-missing items. The range of FI was 0-1, and FI >0.21 was considered to indicate frailty.

Although FI was calculated as a continuous deficit accumulation score, frailty status was defined using the established cutoff of FI >0.21. This dichotomized outcome was selected as the primary endpoint because the present study focused on clinically meaningful frailty rather than small continuous changes in FI. Using a common cutoff also allowed consistent classification of frailty across NHANES and UKB and facilitated interpretation of the results as odds ratios for frailty.

#### Assessment of covariates

The following covariates were included in the fully adjusted model. Continuous covariates included chronological age, energy intake, total fat intake, protein intake, carbohydrate intake, neutrophil count, and lymphocyte count. Categorical covariates included sex, race/ethnicity, educational level, and poverty. Race/ethnicity was defined according to the categories available in each dataset. In NHANES, race/ethnicity was categorized as Mexican American, Non-Hispanic Black, Non-Hispanic White, and Other Race. In UKB, race/ethnicity was categorized as White and Other. Educational level was categorized as less than high school, high school or equivalent, and college or above. Poverty was categorized as yes or no.

### Statistical analysis

#### MR analysis

All MR analyses relied on three core assumptions: the genetic variants are associated with AFS, are not associated with confounders of the AFS-frailty association, and affect frailty only through AFS.^21^ The inverse variance weighted (IVW) method was used as the main MR method.^22^ When heterogeneity was detected, a random-effects IVW model was used; otherwise, a fixed-effects IVW model was applied.^23^ MR Egger, weighted median, simple model, and weighted model methods were used as supplementary MR approaches. The process of selecting instrumental variables (IV) is described in Supplementary Methods.

To evaluate the robustness of the MR findings, heterogeneity was assessed using Cochran’s Q test, with *P* < .05 and I^2^ > 25% indicating the presence of heterogeneity. Horizontal pleiotropy was assessed using the MR-Egger intercept. Leave-one-out (LOO) analysis was performed to examine whether the MR estimates were driven by a single genetic variant. Because AFS and frailty GWAS partly included UKB participants, sample overlap and winner’s curse were assessed using an overlap calculator and MRlap correction.^24^

#### Population-based observational analyses

Baseline characteristics were summarized across quintiles of AFS among participants with AFS ≥15 years. In NHANES, oversampling, stratification, and clustering were accounted for to obtain nationally representative estimates. Continuous variables were presented as means with SD, and categorical variables were summarized as numbers and percentages. Differences across groups were tested using analysis of variance for continuous variables and chi-square tests for categorical variables. For NHANES, survey-weighted methods and Rao-Scott chi-square tests were used to account for the complex survey design. Participants with missing AFS or frailty data were excluded before imputation. Missing covariate data were handled using Multiple Imputation by Chained Equations (MICE), performed separately for NHANES and UKB.

To evaluate the association between AFS and frailty, analyses proceeded in three steps. First, RCS functions within logistic regression models were used to assess potential nonlinearity between AFS and frailty among participants with AFS ≥15 years. Second, when nonlinearity was detected, the derivative method was used to identify the inflection point of the fitted RCS curve. These inflection points typically corresponded to locations with the smallest absolute derivative or the largest change in the derivative. We then fitted two-piecewise logistic regression models on either side of the inflection point.^25^ The threshold was selected as the inflection point that maximized the model likelihood, and log-likelihood ratio tests were used to compare the single-line and two-piecewise models.^25^ Logistic regression models were applied to evaluate the threshold effect of AFS on frailty risk. Third, logistic regression models were used to evaluate the association between AFS quintiles and frailty, with the third quintile as the reference group. Participants reporting AFS <15 years were analyzed separately and were not included in the quintile categories. Sex-stratified analyses were also performed, and sex was not included as a covariate in these models. Because multiple secondary analyses were performed across cohorts, sex strata, AFS quintiles, and threshold models, the false discovery rate (FDR) was controlled using the Benjamini-Hochberg method. All analyses were performed using R software version 4.5.0 (R Foundation for Statistical Computing, Vienna, Austria). This study was based on secondary analyses of existing population-based datasets and publicly available GWAS summary statistics; therefore, no study-specific clinical devices or laboratory instruments were used by the authors.

## Results

### Baseline characteristics of study participants in the population-based observational analyses

After exclusions, 8282 participants from NHANES and 109 449 participants from UKB were included in the primary observational analyses among participants with AFS ≥15 years. In NHANES, 46% of participants were male, and in UKB, 44% were male. Table 1 shows the baseline characteristics of participants according to AFS quintiles. The quintile ranges for AFS were 15-16, 17, 18-19, 20-21, and 22-68 years in NHANES, and 15-17, 18, 19-20, 21-22, and 23-65 years in UKB. In NHANES, baseline characteristics differed significantly across AFS quintiles, except for protein intake and carbohydrate intake. In UKB, all baseline characteristics differed significantly across AFS quintiles.

**Table 1:** Baseline characteristics of participants from US National Health and Nutrition Examination Survey (NHANES) and UK Biobank according to the quintiles of age at first sexual intercourse (AFS).

US NHANES										UK Biobank					
Characteristics	Total population (*N* = 8282)	AFS:15-16 years (*N* = 2234)	AFS:17 years (*N* = 1218)	AFS:18-19 years (*N* = 2222)	AFS:20-21 years (*N* = 1232)	AFS:22-68 years (*N* = 1376)	*P* value		Total population (*N* = 109 449)	AFS: 15-17 years (*N* = 29 631)	AFS: 18 years (*N* = 17 352)	AFS: 19-20 years (*N* = 24 643)	AFS: 21-22 years (*N* = 18 430)	AFS: 23-65 years (*N* = 19 393)	*P* value
**Age**	58.0 (5.56)	57.6 (5.45)	57.7 (5.46)	58.1 (5.59)	58.7 (5.65)	58.5 (5.59)	<.001		59.13 (5.21)	57.52 (4.97)	58.31 (5.03)	59.25 (5.01)	60.38 (5.00)	60.97 (5.26)	<.001
**Gender**							<.001								<.001
Female	4454 (54%)	1045 (47%)	643 (53%)	1220 (55%)	719 (58%)	827 (60%)			61 212 (56%)	17 294 (58%)	9836 (57%)	14 426 (59%)	10 493 (57%)	9163 (47%)	
Male	3828 (46%)	1189 (53%)	575 (47%)	1002 (45%)	513 (42%)	549 (40%)			48 237 (44%)	12 337 (42%)	7516 (43%)	10 217 (41%)	7937 (43%)	10 230 (53%)	
**Education level**							<.001								<.001
College or above	6432 (78%)	1598 (72%)	952 (78%)	1778 (80%)	1023 (83%)	1081 (79%)			49 207 (45%)	9992 (34%)	7515 (43%)	11 854 (48%)	9196 (50%)	10 650 (55%)	
High school or equivalent	1072 (13%)	410 (18%)	166 (14%)	267 (12%)	92 (7%)	137 (10%)			35 854 (33%)	10 980 (37%)	5902 (34%)	7802 (32%)	5689 (31%)	5481 (28%)	
Less than high school	778 (9%)	226 (10%)	100 (8%)	177 (8%)	117 (10%)	158 (11%)			24 388 (22%)	8659 (29%)	3935 (23%)	4987 (20%)	3545 (19%)	3262 (17%)	
**Race/ethnicity**							<.001								<.001
Mexican American	1235 (15%)	308 (14%)	187 (15%)	298 (13%)	196 (16%)	246 (18%)									
Non-Hispanic Black	1737 (21%)	643 (29%)	276 (23%)	470 (21%)	183 (15%)	165 (12%)									
Non-Hispanic White/White	3806 (46%)	978 (44%)	593 (49%)	1106 (50%)	613 (50%)	516 (38%)			107 093 (98%)	29 039 (98%)	17 038 (98%)	24 170 (98%)	18 070 (98%)	18 776 (97%)	
Other Race	1504 (18%)	305 (13%)	162 (13%)	348 (16%)	240 (19%)	449 (32%)			2356 (2%)	592 (2%)	314 (2%)	473 (2%)	360 (2%)	617 (3%)	
**Poverty**							<.001								<.001
No	7105 (86%)	1826 (82%)	1034 (85%)	1942 (87%)	1094 (89%)	1209 (88%)			92 470 (84%)	24 796 (84%)	14 698 (85%)	20 978 (85%)	15 562 (84%)	16 436 (85%)	
Yes	1177 (14%)	408 (18%)	184 (15%)	280 (13%)	138 (11%)	167 (12%)			16 979 (16%)	4835 (16%)	2654 (15%)	3665 (15%)	2868 (16%)	2957 (15%)	
**Neutrophils**	4.10 (1.57)	4.17 (1.63)	4.14 (1.64)	4.13 (1.59)	4.08 (1.53)	3.93 (1.42)	<.001		4.05 (1.26)	4.10 (1.31)	4.03 (1.27)	4.02 (1.25)	4.02 (1.24)	4.04 (1.24)	<.001
**Lymphocyte**	2.13 (1.01)	2.18 (0.82)	2.20 (1.72)	2.11 (0.75)	2.07 (1.04)	2.05 (0.68)	<.001		1.93 (1.06)	1.99 (1.33)	1.94 (0.72)	1.93 (1.10)	1.91 (0.87)	1.87 (0.98)	<.001
**Energy intake**	2015 (875)	2086 (948)	2021 (883)	2020 (880)	1952 (811)	1944 (774)	<.001		8686.68 (1166.46)	8664.19 (1200.15)	8695.38 (1203.48)	8676.93 (1182.94)	8694.30 (1097.75)	8718.42 (1121.27)	<.001
**Protein intake**	78.7 (38.6)	80.5 (42.3)	78.9 (39.2)	78.3 (37.5)	76.9 (34.8)	77.6 (36.4)	.072		80.92 (11.54)	80.78 (11.91)	80.91 (11.97)	80.87 (11.61)	80.99 (10.97)	81.13 (11.01)	.014
**Total fat intake**	77.9 (43.3)	81.9 (45.9)	79.0 (44.1)	78.2 (43.8)	75.2 (41.2)	72.7 (38.4)	<.001		73.09 (13.97)	72.75 (14.06)	73.18 (14.66)	73.03 (14.22)	73.27 (13.39)	73.46 (13.42)	<.001
**Carbohydrate intake**	241 (110)	241 (113)	239 (111)	242 (111)	237 (109)	242 (102)	.664		256.16 (36.81)	255.14 (37.74)	255.85 (36.27)	255.82 (37.30)	256.59 (35.24)	258.00 (36.63)	<.001
**Frailty**							<.001								<.001
Yes	2139 (26%)	769 (34%)	332 (27%)	521 (23%)	265 (22%)	252 (18%)			2001 (2%)	715 (2%)	297 (2%)	401 (2%)	287 (2%)	301 (2%)	
No	6143 (74%)	1465 (66%)	886 (73%)	1701 (77%)	967 (78%)	1124 (82%)			107 448 (98%)	28 916 (98%)	17 055 (98%)	24 242 (98%)	18 143 (98%)	19 092 (98%)	

In US NHANES, all estimates accounted for complex survey designs, and *P* values were calculated using analysis of variance adjusting for sampling weights and Rao-Scott χ2

Test for continuous and categorical variables, respectively. In UK Biobank, *P* values were calculated using analysis of variance and χ2 test for continuous and categorical variables, respectively. Continuous variables were presented as mean ± SD, and categorical variables were presented as percentages.

Race/ethnicity categories were defined separately according to the available classifications in each dataset. NHANES race/ethnicity categories included Mexican American, Non-Hispanic Black, Non-Hispanic White, and Other Race. UK Biobank race/ethnicity categories included White and Other; US-specific categories were not applicable to UK Biobank.

### MR analysis of AFS and frailty

**Figure 1:**
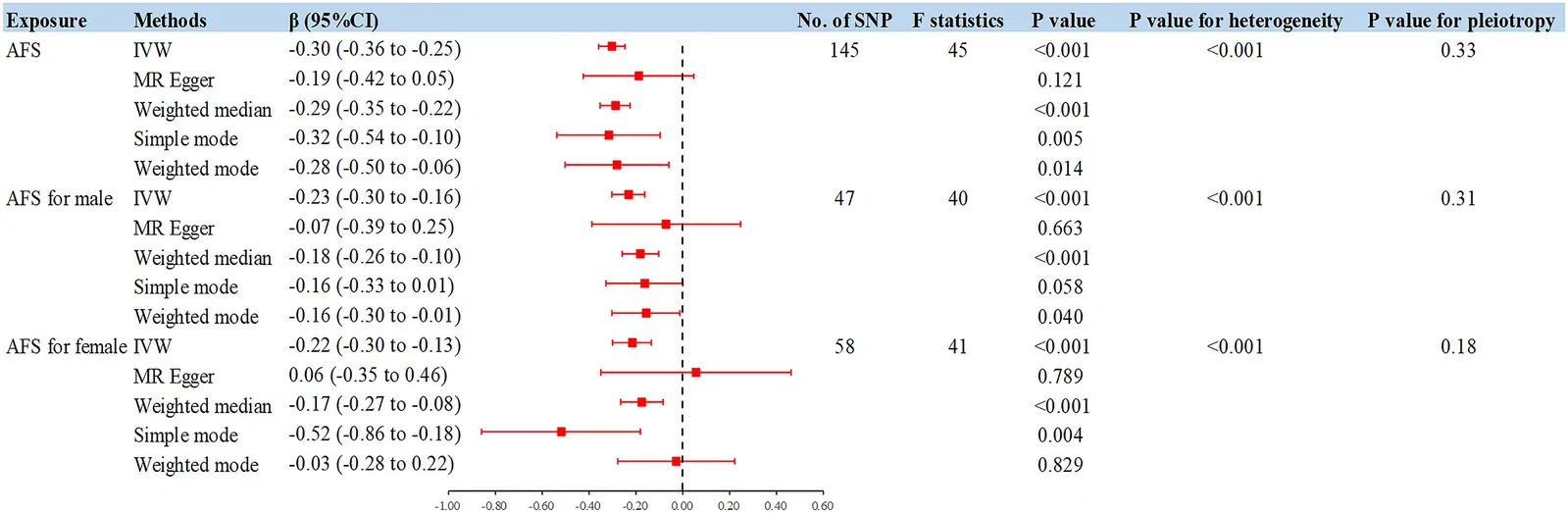
Mendelian randomization estimates for the causal associations between AFS and frailty.

The IVW analysis indicated that genetically predicted earlier AFS was associated with higher risk of frailty (β = -0.30; 95% CI, -0.36 to -0.25), with similar estimates in males (β = -0.23; 95% CI, -0.30 to -0.16) and females (β = -0.22; 95% CI, -0.30 to -0.13) (Figure 1). MR-Egger intercepts suggested no directional horizontal pleiotropy (P > 0.05). Heterogeneity was present (*P* < .001), but instrument strength was adequate (all F statistics ≥40), and LOO analyses identified no influential outlier SNPs (Supplementary Figures 3 and 4). Sample-overlap type I error was controlled below 0.05 (Supplementary Table 1), and MRlap-corrected estimates were close to uncorrected estimates (Supplementary Table 2), suggesting limited overlap-induced bias relative to sampling variability. This limited change is plausible given the adequate strength of the genetic instruments.

### Association of AFS and risks of frailty

**Figure 2:**
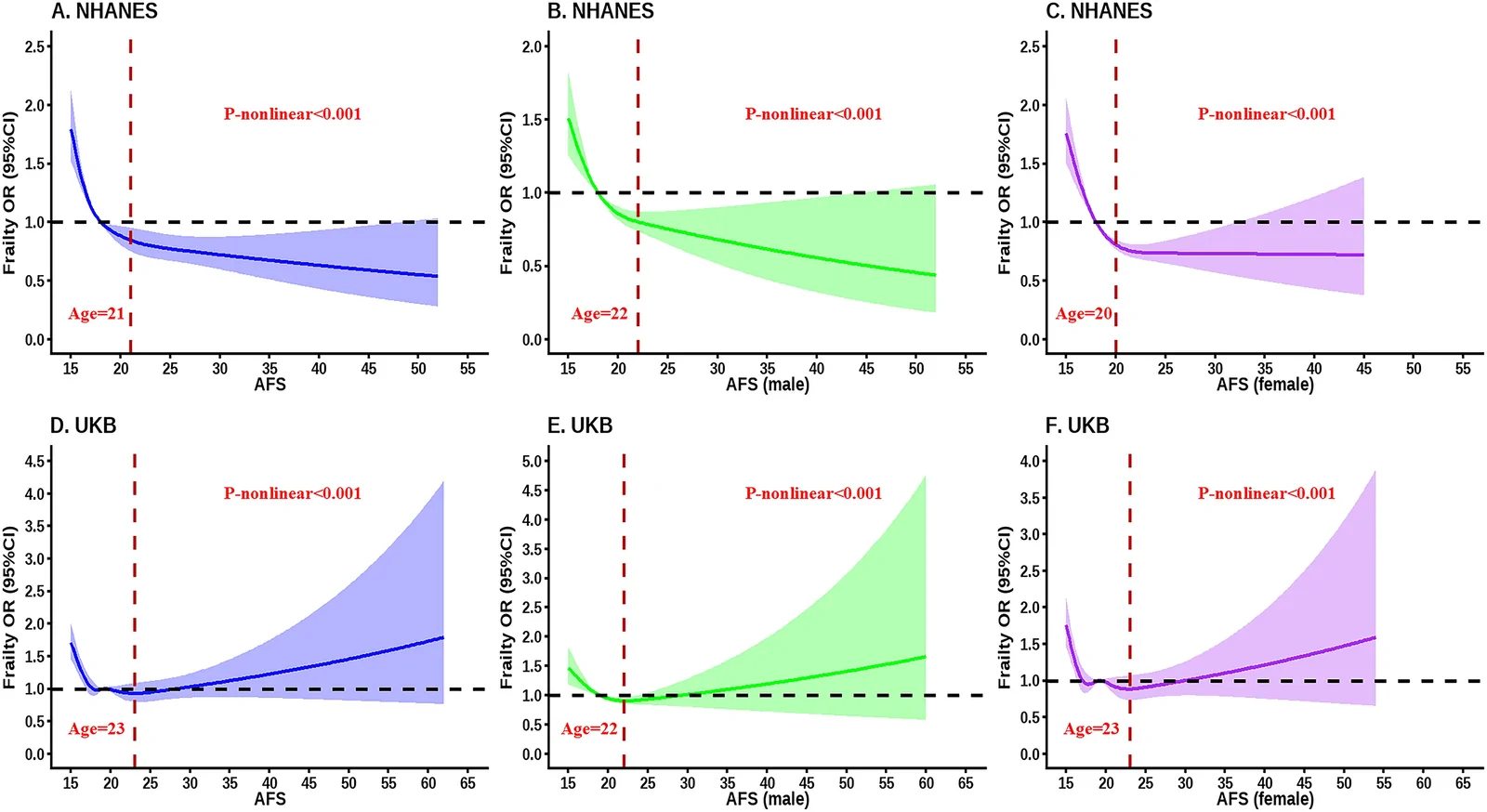
Restricted cubic spline fitting plot of the nonlinear relationship between AFS and frailty.

To harmonize the analytic range across cohorts and to avoid conflating very early reported sexual experiences with ordinary sexual initiation, participants reporting AFS <15 years were analyzed separately, while the quintile-based analyses were conducted among participants with AFS ≥15 years. RCS analyses showed a significant nonlinear relationship between AFS and frailty in both NHANES and UKB, with an L-shaped pattern in the overall sample as well as in males and females (*P* for nonlinearity <.001; Figure 2). In the fully adjusted overall model, AFS <15 years was not significantly associated with frailty in either NHANES (OR = 0.94, 95% CI, 0.88-1.01) or UKB (OR = 1.04, 95% CI, 0.80-1.37) (Table 1). In the quintile-based analyses, younger AFS remained associated with higher odds of frailty. Compared with Q3, participants in Q1 had higher odds of frailty in both NHANES (OR = 1.61, 95% CI, 1.40-1.85) and UKB (OR = 1.34, 95% CI, 1.18-1.52) after full adjustment. In NHANES, Q2 was also associated with higher odds of frailty (OR = 1.19, 95% CI, 1.05-1.41), whereas Q5 was associated with lower odds of frailty (OR = 0.77, 95% CI, 0.64-0.91). In UKB, the associations for Q2, Q4, and Q5 were not statistically significant in the fully adjusted model (Table 2).

**Table 2:** Association of AFS and risks of frailty.

Exposure	US NHANES				UK Biobank			
	Model 1	Model 2	Model 3	Model 4	Model 1	Model 2	Model 3	Model 4
**AFS**								
<15 years	0.95 (0.89-1.01)	0.94 (0.88-1.01)	0.95 (0.88-1.01)	0.94 (0.88-1.01)	0.98 (0.76-1.28)	0.98 (0.77-1.30)	1.04 (0.80-1.37)	1.04 (0.80-1.37)
Q1	1.71 (1.50-1.95)^***^	1.75 (1.53-2.01)^***^	1.62 (1.41-1.85)^***^	1.61 (1.40-1.85)^***^	1.49 (1.32-1.69)^***^	1.57 (1.38-1.78)^***^	1.37 (1.21-1.55)^***^	1.34 (1.18-1.52)^***^
Q2	1.22 (1.04-1.43)^**^	1.23 (1.04-1.44)^**^	1.20 (1.02-1.41)^**^	1.19 (1.05-1.41)^**^	1.05 (0.90-1.22)	1.09 (0.94-1.27)	1.03 (0.89-1.20)	1.03 (0.89-1.21)
Q3	Ref	Ref	Ref	Ref	Ref	Ref	Ref	Ref
Q4	0.89 (0.76-1.06)	0.89 (0.75-1.05)	0.92 (0.78-1.09)	0.94 (0.79-1.11)	0.96 (0.82-1.11)	0.94 (0.80-1.09)	0.96 (0.83-1.12)	0.97 (0.83-1.13)
Q5	0.73 (0.62-0.87)^***^	0.73 (0.61-0.87)^***^	0.74 (0.62-0.88)^***^	0.77 (0.64-0.91)^**^	0.95 (0.83-1.11)	0.96 (0.82-1.12)	1.03 (0.89-1.20)	1.04 (0.89-1.21)
*P* for trend	<.001	<.001	<.001	<.001	<.001	<.001	<.001	<.001
**AFS (male)**								
<15 years	0.93 (0.85-1.01)	0.93 (0.86-1.02)	0.94 (0.87-1.03)	0.94 (0.86-1.03)	1.40 (0.98-2.07)	1.42 (1.04-2.10)^***^	1.48 (1.04-2.21)^***^	1.46 (1.02-2.19)^***^
Q1	1.62 (1.33-1.98)^***^	1.58 (1.29-1.94)^***^	1.50 (1.22-1.84)^***^	1.49 (1.21-1.83)^***^	1.84 (1.45-2.35)^***^	1.90 (1.45-2.44)^***^	1.51 (1.19-1.94)^***^	1.47 (1.15-1.89)^**^
Q2	1.01 (0.78-1.30)	1.02 (0.79-1.32)	1.02 (0.78-1.32)	1.01 (0.78-1.32)	1.32 (0.98-1.76)	1.35 (1.01-1.79)^*^	1.24 (0.93-1.65)	1.23 (0.92-1.64)
Q3	Ref	Ref	Ref	Ref	Ref	Ref	Ref	Ref
Q4	1.03 (0.79-1.33)	1.05 (0.80-1.36)	1.08 (0.82-1.41)	1.09 (0.82-1.42)	1.06 (0.79-1.43)	1.04 (0.77-1.40)	1.08 (0.80-1.46)	1.09 (0.80-1.47)
Q5	0.61 (0.45-0.81)^***^	0.63 (0.47 to 0.84)^***^	0.63 (0.46-0.84)^**^	0.65 (0.48-0.88)^**^	1.18 (0.90-1.55)	1.10 (0.84-1.45)	1.17 (0.89-1.55)	1.17 (0.89- 1.55)
* P* for trend	<.001	<.001	<.001	<.001	<.001	<.001	<.001	<.001
**AFS (Female)**								
<15 years	0.95 (0.84-1.06)	0.95 (0.85- 1.06)	0.95 (0.84-1.07)	0.94 (0.83-1.07)	0.58 (0.40-0.86)^***^	0.59 (0.41-0.88)^***^	0.63 (0.43-.95)^**^	0.62 (0.42-0.93)^**^
Q1	1.93 (1.62-2.30)^***^	1.89 (1.59- 2.27)^***^	1.71 (1.43- 2.06)^***^	1.72 (1.43- 2.07)^***^	1.39 (1.21-1.60)^***^	1.45 (1.25-1.68)^***^	1.30 (1.13-1.51)^***^	1.28 (1.10-1.49)^***^
Q2	1.42 (1.16-1.75)^***^	1.41 (1.14-1.73)^**^	1.35 (1.09-1.67)^**^	1.35 (1.08-1.67)^**^	0.98 (0.82-1.17)	1.01 (0.84-1.20)	0.96 (0.80-1.15)	0.96 (0.80-1.15)
Q3	Ref	Ref	Ref	Ref	Ref	Ref	Ref	Ref
Q4	0.81(0.65-1.03)	0.81 (0.65-1.03)	0.84 (0.67-1.05)	0.86 (0.69-1.08)	0.93 (0.78-1.12)	0.91 (0.76-1.08)	0.93 (0.77-1.11)	0.93 (0.78-1.12)
Q5	0.78 (0.63-0.96)^***^	0.78 (0.63-0.97)^*^	0.81 (0.65-1.01)	0.84 (0.67-1.04)	0.97 (0.80-1.16)	0.92 (0.76-1.10)	0.98 (0.81-1.18)	0.98 (0.81-1.18)
* P* for trend	<.001	<.001	<.001	<.001	<.001	<.001	<.001	.004

Analysis in US NHANES included the US population and study design weights to account for the complex survey design. Participants reporting AFS <15 years were analyzed separately and were not included in the quintile categories. Quintiles were defined among participants with AFS ≥15 years. In NHANES, Q1 = 15-16 years, Q2 = 17 years, Q3 = 18-19 years, Q4 = 20-21 years, and Q5 = 22-68 years. In UK Biobank, Q1 = 15-17 years, Q2 = 18 years, Q3 = 19-20 years, Q4 = 21-22 years, and Q5 = 23-65 years. OR (95% confidence interval) and p-value were presented in the table.^***^P < .001,^**^P < .01,^*^P < .05

Model 1 = no covariates were adjusted.

Model 2 = age, sex, and race/ethnicity were adjusted.

Model 3 = age, sex, race/ethnicity, educational level, and poverty were adjusted.

Model 4 = age, sex, race/ethnicity, educational level, poverty, protein intake, energy intake, total fat intake, carbohydrate intake, neutrophil count, and lymphocyte count were adjusted.

In sex-stratified analyses, sex was not included as a covariate.

Abbreviations: AFS, age at first sexual intercourse; US NHANES, US National Health and Nutrition Examination Survey

Sex-stratified analyses showed a generally similar pattern, although some differences were observed between males and females. In NHANES, Q1 was associated with higher odds of frailty in both males (OR = 1.49, 95% CI, 1.21-1.83) and females (OR = 1.72, 95% CI, 1.43-2.07) after full adjustment. Q2 was significantly associated with higher odds of frailty among females (OR = 1.35, 95% CI, 1.08-1.67), but not among males. Conversely, Q5 was associated with lower odds of frailty among males (OR = 0.65, 95% CI, 0.48-0.88), whereas this association was not statistically significant among females. In UKB, Q1 was also associated with higher odds of frailty in both males (OR = 1.47, 95% CI, 1.15-1.89) and females (OR = 1.28, 95% CI, 1.10-1.49), while the associations for Q2, Q4, and Q5 were not statistically significant in either sex. The estimates for AFS <15 years differed by sex in UKB and should be interpreted cautiously because very early reported AFS may reflect heterogeneous contextual experiences.

### Threshold effects analysis between AFS and frailty

We next examined whether the association between AFS and frailty showed threshold effects. Inflection points were first estimated as continuous values using the derivative method based on the RCS curves and were then operationalized as integer age cut points for the two-piecewise logistic regression models, because AFS was reported in completed years (Supplementary Figure 5). In the overall sample, the integer cut points were 21 years in NHANES and 23 years in UKB. In sex-stratified analyses, the corresponding cut points were 22 years in NHANES males, 20 years in NHANES females, 22 years in UKB males, and 23 years in UKB females. Log-likelihood ratio tests supported the two-piecewise logistic regression model over the single-line model in both datasets and across sex strata (all *P* for log-likelihood ratio tests <.001; Table 3).

**Table 3:** Threshold effect analysis of AFS on frailty.

Database	Cut-off age	Adjusted OR (95% CI) ^a^ , *P* value			
		Model 1	Model 2	Model 3	Model 4
**US NHANES**	Threshold age (both gender)^b^	21	21	21	21
	<21	0.86 (0.83-0.89)^***^	0.85 (0.82-0.88)^***^	0.88 (0.84-0.91)^***^	0.88 (0.85-0.92)^***^
	≥21	0.98 (0.97-1.02)	0.99 (0.98-1.02)	0.99 (0.97-1.02)	0.99 (0.97- 1.01)
	*P* for log likelihood ratio test	<.001	<.001	<.001	<.001
	Threshold age (male)^b^	22	22	22	22
	<22	0.89 (0.85-0.93)^***^	0.90 (0.85-0.94)^***^	0.91 (0.87-0.96)^***^	0.92 (0.87-0.96)^***^
	≥22	1.02 (0.97-1.04)	1.02 (0.97-1.04)	1.01 (0.96-1.04)	1.01 (0.96-1.04)
	*P* for log likelihood ratio test	<.001	<.001	<.001	<.001
	Threshold age (female)^b^	20	20	20	20
	<20	0.79 (0.75-0.84)^***^	0.79 (0.75-0.84)^***^	0.83 (0.78 to 0.88)^***^	0.83 (0.78-0.88)^***^
	≥20	0.99 (0.96-1.02)	0.98 (0.97-1.02)	0.99 (0.96-1.02)	0.99 (0.96-1.02)
	*P* for log likelihood ratio test	<.001	<.001	<.001	<.001
**UK Biobank**	Threshold age (both gender)^b^	23	23	23	23
	<23	0.90 (0.87-0.92)^***^	0.88 (0.86-0.90)^***^	0.92 (0.89-0.94)^***^	0.92 (0.90-0.94)^***^
	≥23	1.03 (0.96-1.06)	1.02 (0.99-1.04)	1.01 (0.98-1.04)	1.01 (0.98-1.03)
	*P* for log likelihood ratio test	<0.001	<0.001	<0.001	<0.001
	Threshold age (male)^b^	22	22	22	22
	<22	0.87 (0.82-0.91)^***^	0.86 (0.81-0.90)^***^	0.92 (0.87-0.96)^***^	0.92 (0.87-0.97)^***^
	≥22	1.02 (0.98-1.05)	1.02 (0.97-1.05)	1.01 (0.98- 1.04)	1.03 (0.97-1.06)
	*P* for log likelihood ratio test	<.001	<.001	<.001	<.001
	Threshold age (female)^b^	23	23	23	23
	<23	0.91 (0.88-0.94)^***^	0.89 (0.87-0.92)^***^	0.92 (0.89-0.95)^***^	0.92 (0.89-0.95)^***^
	≥23	1.02 (0.98-1.05)	1.02 (0.98-1.05)	1.01 (0.98-1.05)	1.01 (0.98-1.05)
	*P* for log likelihood ratio test	<.001	<.001	<.001	<.001

Analysis in US NHANES included the US population and study design weights to account for the complex survey design. OR (95% CI) and *P*-value were presented in the table.

Thresholds were estimated as continuous inflection points using the derivative method based on restricted cubic spline curves and were then operationalized as integer age cut points for the two-piecewise logistic regression models because AFS was reported in completed years. Continuous estimates are shown in Supplementary Figure 5.

Model 1 = no covariates were adjusted.

Model 2 = age, sex, and race/ethnicity were adjusted.

Model 3 = age, sex, race/ethnicity, educational level, and poverty were adjusted.

Model 4 = age, sex, race/ethnicity, educational level, poverty, protein intake, energy intake, total fat intake, carbohydrate intake, neutrophil count, and lymphocyte count were adjusted.

In sex-stratified analyses, sex was not included as a covariate.

Abbreviations: AFS, age at first sexual intercourse; OR, odds ratio; 95 % CI, 95 % confidence interval; US NHANES, US National Health and Nutrition Examination Survey

^a^ logistic regression models were used to estimate ORs and 95% CIs. ^b^ Fitting model by 2-piecewise logistic regression models. ^***^ *P* < .001,^**^P < .01,^*^P < .05

In the fully adjusted model, later AFS was associated with lower odds of frailty only below the estimated thresholds. In NHANES, each 1-year increase in AFS before age 21 was associated with 12% lower odds of frailty in the overall sample (OR = 0.88, 95% CI, 0.85-0.92). Similar inverse associations were observed below the thresholds in males before age 22 (OR = 0.92, 95% CI, 0.87-0.96) and females before age 20 (OR = 0.83, 95% CI, 0.78-0.88). In UKB, each 1-year increase in AFS before age 23 was associated with 8% lower odds of frailty in the overall sample (OR = 0.92, 95% CI, 0.90-0.94). The corresponding estimates were also significant in males before age 22 (OR = 0.92, 95% CI, 0.87-0.97) and females before age 23 (OR = 0.92, 95% CI, 0.89-0.95). Above the estimated thresholds, AFS was not significantly associated with frailty in either the overall or sex-stratified analyses. These findings suggest that the inverse association between AFS and frailty was mainly concentrated at younger ages of first sexual intercourse and tended to plateau after the threshold ages.

### Multiple testing correction

To account for multiple testing in secondary and exploratory analyses, we applied Benjamini-Hochberg FDR correction within prespecified families of related tests, including RCS nonlinearity tests, quintile-based logistic regression analyses, and two-piecewise threshold analyses. Both raw and FDR-adjusted P values are reported in Supplementary Table 3. The main findings, including the nonlinear associations, the higher frailty odds in the youngest AFS quintile, and the inverse associations below the estimated thresholds, remained robust after FDR correction.

## Discussion

In this study, MR analyses showed that genetically predicted earlier AFS was associated with higher frailty risk. Similar MR estimates were observed in males and females. In parallel, population-based observational analyses in NHANES and UKB showed an L-shaped association between AFS and frailty among middle-aged and older adults. Frailty risk was higher at younger AFS, declined as AFS increased, and tended to plateau beyond the threshold range, with inflection points of 21 years in NHANES and 23 years in UKB. Overall, these findings suggest a robust association between earlier sexual debut and higher frailty risk later in life, while also suggesting that the strength of this relationship varies across age ranges rather than following a simple linear pattern.

The MR findings are important because they provide genetic evidence supporting a potential relationship between AFS and frailty. This interpretation is supported by the consistency of the IVW estimates with at least one additional MR method, the absence of evidence of pleiotropy, and the adequate strength of the IVs. Our findings extend prior MR evidence that considered AFS within a broader set of female reproductive traits by showing that AFS, when examined as an independent indicator of sexual debut timing, was associated with frailty and that this association is not limited to women.^14^ The broadly similar MR estimates observed in males and females suggest that the relevance of AFS to frailty may extend beyond a narrowly female reproductive framework and may instead reflect broader developmental, behavioral, and psychosocial pathways operating across the life course. Nevertheless, because the GWAS datasets partly relied on UKB participants, these MR findings should be interpreted as supportive genetic evidence rather than definitive proof of causality.

The observational analyses further showed that the association between AFS and frailty was distinctly nonlinear. In both NHANES and UKB, younger AFS was associated with higher frailty risk, whereas the magnitude of association diminished as AFS increased, producing a consistent L-shaped pattern in the overall sample and in both sexes. This finding suggests that the health implications of sexual debut timing are not uniform across age ranges. A plausible explanation is that younger AFS may be more likely to co-occur with a range of adverse experiences and cumulative disadvantages across the life course, including unintended pregnancy,^2^ sexually transmitted infections,^4^ depression and stigma,^3^ educational disruption,^26^ constrained social resources,^1^ and childhood sexual abuse.^27^ Such exposures may cluster within broader trajectories of psychosocial and socioeconomic disadvantage, which have themselves been linked to higher frailty risk later in life.^28^^,^^29^ By contrast, when sexual debut occurs at older ages, individuals may have greater psychosocial maturity, more stable relationships, and better access to protective resources, which may partly attenuate the association between AFS and frailty.^30^ This pattern also suggests that later AFS may represent a distinct developmental pathway that warrants further investigation.

The findings for AFS <15 years should be interpreted cautiously. Although AFS <15 years was not significantly associated with frailty in the overall analyses, sex-stratified estimates in UKB were heterogeneous. Very early reported AFS may not represent the lower end of consensual sexual initiation, but may instead reflect childhood sexual abuse, coercion, age-discordant relationships, or other adverse contexts. Previous studies have linked childhood sexual abuse and adverse childhood experiences to long-term physical and psychological consequences, biological embedding of stress, accelerated aging markers, and later-life frailty.^31^^,^^32^ Therefore, the nonsignificant overall association for AFS <15 years may reflect exposure heterogeneity, differential recall and reporting, limited subgroup size, or selection processes rather than absence of harm. Because neither cohort captured consent, coercion, partner age, or relationship context at first intercourse, we analyzed AFS <15 years separately and interpreted these estimates cautiously.

The threshold analyses further refined this pattern by identifying inflection points at 21 years in NHANES and 23 years in UKB, with modest variation by sex across datasets. Below these thresholds, later AFS was associated with lower odds of frailty, whereas above the threshold the association was no longer statistically significant. These findings suggest that frailty risk is more strongly concentrated among individuals with younger AFS and becomes less sensitive to differences in AFS once sexual debut occurs beyond late adolescence or early adulthood. Importantly, these threshold ages should not be interpreted as defining an ideal or recommended age for first sex. Rather, they should be understood as statistical points at which the magnitude of association changes. This interpretation is also consistent with previous work showing that the implications of early sexual debut depend importantly on the context of first sex, including consent, protection, and broader social support, rather than age alone.^33^^,^^34^

Public health implications should therefore emphasize safe, informed, and rights-based sexual development rather than delaying sexual debut to a specific age. Because younger AFS may be intertwined with social disadvantage, educational disruption, and limited protective resources, interventions that improve early-life environments may also support healthy aging. Relevant strategies include comprehensive sexuality education emphasizing consent and healthy relationships, and equitable access to sexual and reproductive health services. In addition, the fact that the inflection points differed somewhat between NHANES and UKB and varied modestly by sex suggests that these thresholds should not be translated into universal policy targets. Instead, sexual-health strategies should be interpreted within their specific social and cultural contexts, and future work in more diverse settings will be needed to clarify the generalizability of these patterns.

This study has several strengths. To our knowledge, it is the first to combine MR with two large population-based datasets to examine both genetic evidence and observational patterns linking AFS with frailty. The identification of threshold ages further adds precision to the interpretation of the observed association. In addition, the consistency of findings across MR, NHANES, and UKB strengthens confidence in the overall pattern. Several limitations should also be acknowledged. First, the observational analyses based on NHANES and UKB were cross-sectional and therefore cannot establish temporality or prove causality. Reverse causation and residual confounding cannot be excluded. Second, AFS was self-reported, and participants with poorer current health or frailty may recall or report earlier-life sexual experiences differently, introducing potential recall bias. Third, AFS captures the timing of first sex only and does not provide information on consent, coercion, relationship context, or protection. Therefore, AFS <15 years was not treated as part of the ordinary AFS quintile distribution. Nevertheless, residual exposure misclassification remains possible because neither NHANES nor UKB collected detailed contextual information on first sex. Fourth, potential sample overlap remains a limitation of the MR analyses because both the AFS and frailty GWAS included UKB participants. Although overlap-related bias was quantified and MRlap correction yielded estimates close to the original results, the MR findings should be interpreted cautiously. Finally, UKB is subject to healthy volunteer selection, and MR analyses were conducted primarily in individuals of European ancestry; therefore, generalizability to other populations and sociocultural contexts remains uncertain.

## Conclusion

MR analyses provided genetic evidence consistent with a potential association between earlier AFS and higher frailty risk. NHANES and UKB analyses showed a consistent L-shaped association among middle-aged and older adults, concentrated at younger AFS and plateauing beyond threshold ranges. However, given the cross-sectional observational design, residual confounding, reverse causation, recall bias, and the lack of contextual information on first sex, these findings should be interpreted cautiously and should not be regarded as definitive evidence of causality or as indicating an optimal age for sexual debut.

## Supplementary Material

Supplementary_material_qfag075

## Data Availability

US NHANES data could be obtained on application from https://www.cdc.gov/nchs/nhanes/. UKB data could be obtained on application from https://www.ukbiobank.ac.uk/enable-your-research/apply-for-access.
