# Supplementary material for: Age at first sexual intercourse and frailty: Mendelian randomization and population-based evidence from NHANES and UK Biobank
Source: Sex Med. 2026 Sep 2;14(6):qfag075. doi: 10.1093/sexmed/qfag075 (PMC13537451; doi:10.1093/sexmed/qfag075)
Supplement: Supplementary file 1 [file supplementary_material_qfag075.zip › Supplementary Material.docx]

**Supplementary Material 1**

**Supplementary Method.** The progress of selection of genetic instruments and MR sensitivity analysis

**Supplementary Figure 1.** Flow chart of US NHANES
**Supplementary Figure 2.** Flow chart of UK Biobank
**Supplementary Figure 3.** MR scatter plot for the causal associations of AFS with frailty
**Supplementary Figure 4.** MR leave-one out plots of AFS on frailty
**Supplementary Figure 5.** The first derivative of restrictive cubic spline

**Supplementary Table 1.** Type 1 error rate for the causal estimates due to sample overlap between AFS and frailty

**Supplementary Table 2.** MRlap estimates for the causal associations between AFS and frailty
**Supplementary Table 3.** Benjamini–Hochberg false discovery rate correction for secondary and exploratory analyses

**Supplementary Method: Selection of genetic instruments**

When selecting IVs in MR analysis, several key principles are followed. First, SNPs robustly associated with AFS were initially screened at genome-wide significance (P < 5 × 10^−8^). The identified SNPs were clumped for LD with a strict cutoff of clumping R^2^ = 0.001 within a window of 10,000 kb, and LD was estimated with the European samples from the 1000 Genome Project as reference^1^. Second, the IVs showing association with the frailty rather than AFS (identified by P < 5 × 10^-8^ in the outcomes) were removed. Third, we also calculated F-statistics for each instrumental variable, retaining only those with an F > 10 to avoid bias caused by weak instruments^2^. F-statistics was calculated by $F=\left[ R^{2}\times\left( N-2 \right) \right]/1-R^{2}$, where $R^{2}=\beta^{2}/\left( \beta^{2}+{SE}^{2}\times N \right)$. N represents the number of participants, and β is the estimated effect of the SNP to assess its ability to predict the outcome uniquely^3,4^. Fourth, palindromic SNPs and incompatible SNPs were removed from IVs after harmonizing exposure and outcome data. Fifth, we utilized MR-PRESSO analysis to remove outlier pleiotropic SNPs (P < 0.05, Nb-Distribution = 1000). Sixth, MR-Steiger filtering was performed to remove reverse causal genetic IVs, which are SNPs that explain more variance in the outcome than in the exposure^5^.After removing reverse causal SNPs, we repeated the MR analyses. Finally, the remaining instrumental variables were included in the analysis.

Reference

1	Auton, A. *et al.* A global reference for human genetic variation. *Nature* **526**, 68-74, doi:10.1038/nature15393 (2015).

2	Bowden, J. & Holmes, M. V. Meta-analysis and Mendelian randomization: A review. *Res Synth Methods* **10**, 486-496, doi:10.1002/jrsm.1346 (2019).

3	Gill, D., Efstathiadou, A., Cawood, K., Tzoulaki, I. & Dehghan, A. Education protects against coronary heart disease and stroke independently of cognitive function: evidence from Mendelian randomization. *Int J Epidemiol* **48**, 1468-1477, doi:10.1093/ije/dyz200 (2019).

4	Levin, M. G. *et al.* Genetics of height and risk of atrial fibrillation: A Mendelian randomization study. *PLoS Med* **17**, e1003288, doi:10.1371/journal.pmed.1003288 (2020).

5	Hemani, G., Tilling, K. & Davey Smith, G. Orienting the causal relationship between imprecisely measured traits using GWAS summary data. *PLoS Genet* **13**, e1007081, doi:10.1371/journal.pgen.1007081 (2017).
**Supplementary Figure 1.** Flow chart of US NHANES

**Supplementary Figure 2.** Flow chart of UK Biobank

**Supplementary Figure 3.** MR scatter plot for the causal associations of AFS with frailty

**Note:** Scatter plot of the effect of each SNP on AFS (male and female) versus frailty index, with the slope of each line corresponding to the estimated MR effect per method. Data are expressed as raw β or OR coefficients with 95% CIs.

**Abbreviations:** AFS, age at first sexual intercourse; CI, confidence interval; IVW, inverse-variance weighted; MR, Mendelian randomization; SNP, single nucleotide polymorphism

A. Genetic association between AFS and frailty

B. Genetic association between AFS for male and frailty

C. Genetic association between AFS for female and frailty

**Supplementary Figure 4.** MR leave-one out plots of AFS on frailty

**Note:** The red lines represent the relationships of AFS (male and female) with frailty using IVW method. The black line is the deviation of the 95% Cl corresponding to the estimate of the SNPs.

**Abbreviations:** AFS, age at first sexual intercourse; CI, confidence interval; IVW, inverse-variance weighted; MR, Mendelian randomization; SNP, single nucleotide polymorphism

A. MR leave-one-out analysis for AFS on frailty

B. MR leave-one-out analysis for AFS of male on frailty

C. MR leave-one-out analysis for AFS of female on frailty

**Supplementary Figure 5.** The first derivative of restrictive cubic spline

Note: Supplementary Figure 5 shows the continuous inflection points estimated using the derivative method based on restricted cubic spline curves. For the two-piecewise logistic regression models shown in Table 3, these continuous estimates were operationalized as integer age cut points because AFS was reported in completed years.

**Supplementary Table 1.** **Type 1 error rate for the causal estimates due to sample overlap between AFS and frailty**

| **Exposures** | **Sample size of exposure GWAS** | **Outcomes** | **Sample size of outcome GWAS** | **Overlap degree** | **Number of instruments** | **R2** | **Observational estimate** | **Bias** | **Type 1 error rate** |
| --- | --- | --- | --- | --- | --- | --- | --- | --- | --- |
| AFS | 397,338 | Frailty index | 175,226 | 41.42% | 145 | 0.016 | 5.29E-08 | 2.12E-08 | 0.05 |
| AFS for male | 182,791 | Frailty index | 175,226 | 90.05% | 47 | 0.011 | 6.60E-08 | 1.83E-08 | 0.05 |
| AFS for female | 214,547 | Frailty index | 175,226 | 76.72% | 58 | 0.012 | 5.30E-08 | 1.54E-08 | 0.05 |

**Supplementary Table 2. MRlap estimates for the causal associations between AFS and frailty**

| **Exposure** | **Outcome** | **Observed β** | **Observed SE** | **Observed P** | **Corrected β** | **Corrected SE** | **Corrected P** | **Test difference** | **P for difference** |
| --- | --- | --- | --- | --- | --- | --- | --- | --- | --- |
| AFS | Frailty index | -0.29 | 0.026 | 3.72E-29 | -0.33 | 0.032 | 4.21E-25 | 6.11 | 1.02E-09 |
| AFS for male | Frailty index | -0.22 | 0.032 | 8.38E-12 | -0.27 | 0.043 | 2.01E-10 | 5.04 | 4.65E-07 |
| AFS for female | Frailty index | -0.20 | 0.038 | 1.62E-07 | -0.25 | 0.051 | 1.37E-06 | 3.65 | 0.0002 |

**Supplementary Table 3. Benjamini–Hochberg false discovery rate correction for secondary and exploratory analyses**

| **Family** | **Cohort** | **Sex** | **Analysis** | **Comparison** | **Raw P value (fill numeric only)** | **FDR-adjusted P value (auto)** | **Significant after FDR (auto)** |
| --- | --- | --- | --- | --- | --- | --- | --- |
| RCS_nonlinearity | NHANES | Overall | Restricted cubic spline | P for nonlinearity | 0.0000 | 0.0000 | **Yes** |
| RCS_nonlinearity | NHANES | Male | Restricted cubic spline | P for nonlinearity | 0.0000 | 0.0000 | **Yes** |
| RCS_nonlinearity | NHANES | Female | Restricted cubic spline | P for nonlinearity | 0.0000 | 0.0000 | **Yes** |
| RCS_nonlinearity | UKB | Overall | Restricted cubic spline | P for nonlinearity | 0.0006 | 0.0006 | **Yes** |
| RCS_nonlinearity | UKB | Male | Restricted cubic spline | P for nonlinearity | 0.0000 | 0.0000 | **Yes** |
| RCS_nonlinearity | UKB | Female | Restricted cubic spline | P for nonlinearity | 0.0000 | 0.0000 | **Yes** |
| Quintile_logistic | NHANES | Overall | Fully adjusted logistic regression | Q1 vs Q3 | 0.0000 | 0.0000 | **Yes** |
| Quintile_logistic | NHANES | Overall | Fully adjusted logistic regression | Q2 vs Q3 | 0.0020 | 0.0080 | **Yes** |
| Quintile_logistic | NHANES | Overall | Fully adjusted logistic regression | Q4 vs Q3 | 0.3560 | 0.4497 | **No** |
| Quintile_logistic | NHANES | Overall | Fully adjusted logistic regression | Q5 vs Q3 | 0.0030 | 0.0080 | **Yes** |
| Quintile_logistic | NHANES | Male | Fully adjusted logistic regression | Q1 vs Q3 | 0.0000 | 0.0002 | **Yes** |
| Quintile_logistic | NHANES | Male | Fully adjusted logistic regression | Q2 vs Q3 | 0.2310 | 0.3261 | **No** |
| Quintile_logistic | NHANES | Male | Fully adjusted logistic regression | Q4 vs Q3 | 0.4560 | 0.5472 | **No** |
| Quintile_logistic | NHANES | Male | Fully adjusted logistic regression | Q5 vs Q3 | 0.0030 | 0.0080 | **Yes** |
| Quintile_logistic | NHANES | Female | Fully adjusted logistic regression | Q1 vs Q3 | 0.0001 | 0.0003 | **Yes** |
| Quintile_logistic | NHANES | Female | Fully adjusted logistic regression | Q2 vs Q3 | 0.0025 | 0.0086 | **Yes** |
| Quintile_logistic | NHANES | Female | Fully adjusted logistic regression | Q4 vs Q3 | 0.0860 | 0.1720 | **No** |
| Quintile_logistic | NHANES | Female | Fully adjusted logistic regression | Q5 vs Q3 | 0.0970 | 0.1791 | **No** |
| Quintile_logistic | UKB | Overall | Fully adjusted logistic regression | Q1 vs Q3 | 0.0002 | 0.0010 | **Yes** |
| Quintile_logistic | UKB | Overall | Fully adjusted logistic regression | Q2 vs Q3 | 0.0830 | 0.1811 | **No** |
| Quintile_logistic | UKB | Overall | Fully adjusted logistic regression | Q4 vs Q3 | 0.1230 | 0.2109 | **No** |
| Quintile_logistic | UKB | Overall | Fully adjusted logistic regression | Q5 vs Q3 | 0.2340 | 0.3120 | **No** |
| Quintile_logistic | UKB | Male | Fully adjusted logistic regression | Q1 vs Q3 | 0.0060 | 0.0144 | **Yes** |
| Quintile_logistic | UKB | Male | Fully adjusted logistic regression | Q2 vs Q3 | 0.2130 | 0.3408 | **No** |
| Quintile_logistic | UKB | Male | Fully adjusted logistic regression | Q4 vs Q3 | 0.5640 | 0.6153 | **No** |
| Quintile_logistic | UKB | Male | Fully adjusted logistic regression | Q5 vs Q3 | 0.5730 | 0.5979 | **No** |
| Quintile_logistic | UKB | Female | Fully adjusted logistic regression | Q1 vs Q3 | 0.0000 | 0.0000 | **Yes** |
| Quintile_logistic | UKB | Female | Fully adjusted logistic regression | Q2 vs Q3 | 0.2150 | 0.3225 | **No** |
| Quintile_logistic | UKB | Female | Fully adjusted logistic regression | Q4 vs Q3 | 0.8950 | 0.8950 | **No** |
| Quintile_logistic | UKB | Female | Fully adjusted logistic regression | Q5 vs Q3 | 0.4670 | 0.5337 | **No** |
| Threshold_piecewise | NHANES | Overall | Two-piecewise logistic regression | Below threshold | 0.0001 | 0.0001 | **Yes** |
| Threshold_piecewise | NHANES | Overall | Two-piecewise logistic regression | Above threshold | 0.2560 | 0.3840 | **No** |
| Threshold_piecewise | NHANES | Male | Two-piecewise logistic regression | Below threshold | 0.0000 | 0.0000 | **Yes** |
| Threshold_piecewise | NHANES | Male | Two-piecewise logistic regression | Above threshold | 0.4590 | 0.5007 | **No** |
| Threshold_piecewise | NHANES | Female | Two-piecewise logistic regression | Below threshold | 0.0000 | 0.0000 | **Yes** |
| Threshold_piecewise | NHANES | Female | Two-piecewise logistic regression | Above threshold | 0.7690 | 0.7690 | **No** |
| Threshold_piecewise | UKB | Overall | Two-piecewise logistic regression | Below threshold | 0.0000 | 0.0000 | **Yes** |
| Threshold_piecewise | UKB | Overall | Two-piecewise logistic regression | Above threshold | 0.4390 | 0.5268 | **No** |
| Threshold_piecewise | UKB | Male | Two-piecewise logistic regression | Below threshold | 0.0001 | 0.0001 | **Yes** |
| Threshold_piecewise | UKB | Male | Two-piecewise logistic regression | Above threshold | 0.1890 | 0.3240 | **No** |
| Threshold_piecewise | UKB | Female | Two-piecewise logistic regression | Below threshold | 0.0000 | 0.0000 | **Yes** |
| Threshold_piecewise | UKB | Female | Two-piecewise logistic regression | Above threshold | 0.3490 | 0.4653 | **No** |

Note: FDR correction was applied within prespecified families of related tests, including restricted cubic spline nonlinearity tests, quintile-based logistic regression analyses, and two-piecewise threshold analyses. Raw P values and FDR-adjusted P values are shown. The overall analyses were considered primary analyses, whereas sex-stratified, quintile-specific, and threshold analyses were considered secondary or exploratory.
